# Simple innovative adaptor to improve genome walking with convenient PCR

**DOI:** 10.1186/s43141-020-00082-2

**Published:** 2020-10-20

**Authors:** Seyedeh-Samira Ashrafmansouri, Hossein Kamaladini, Fatemeh Haddadi, Marie Seidi

**Affiliations:** Department of Biology, Faculty of Sciences, University of Zabol, Zabol, Iran

**Keywords:** Genome walking, Adaptor, Restriction enzymes, PCR, *map*30 gene, pTZ57R plasmid

## Abstract

**Background:**

Various polymerase chain reaction (PCR)-based methods have been applied for the development of genome walking (GW) technique. These methods which could be based on the application of restriction enzymes or primers have various efficiencies to identify the unknown nucleotide sequences. The present study was conducted to design a new innovative double-strand adaptor using *MAP30* gene sequence of *Momordica charantia* plant as a model to improve genome walking with convenient PCR.

**Results:**

The adaptor was designed using multiple restriction sites of *Hind* III, *Bam*H I, *Eco*R I, and *Bgl* II enzymes with no restriction site in a known sequence of the *MAP30* gene. In addition, no modification was required to add phosphate, amine, or other groups to the adaptor, since restriction enzyme digestion of double-strand adaptor provided the 5′ phosphate group. Here, preparation of the phosphate group in the genomic DNA of the plant digestion with restriction enzymes was performed followed by ligation with digested adaptor containing 5′ phosphate group.

**Conclusion:**

PCR was done to amplify the unknown sequence using *MAP30* gene-specific primer and adaptor primer. Results confirmed the ability of the technique for successful identification of the sequence. Consequently, a newly designed adaptor in the developed technique reduced the time and cost of the method compared to the conventional genome walking; also, cloning and culturing of bacterial steps could be eliminated.

## Background

Complete DNA sequencing provides a lot of information about the entire sequence of organisms’ genomic DNA; however, basic methods like convenient genome walking methods are still needed [27]. In fact, genome walking is performed to identify unknown sequences adjacent to known sequences [32] and is very useful when genetic information about sequence analysis of organisms is limited [27]. The technique could be applied for the identification of the transposable elements, insertional mutagenesis ([16]; V [19].), retroviruses, and cloning of multiple genes in order to study their functions. The other applications include identifying promoters and regulatory elements in genomic DNA [9], gap filling in the genomic sequence, and mapping the intron and exon in genetics [3]. The only requirement to start the genome walking is the availability of a part of the known nucleotide sequence of the genome [14]. Genome walking can use polymerase chain reaction for the identification of unknown regions [31]; therefore, based on the purpose of research, different PCRs are used.

Genome walking can be depended on restriction and ligation reaction such as inverted PCR [29], rolling circle inverted PCR [30], step down PCR [35], cassette ligation [22], and rapid amplification of genomic ends (RAGE) [4]. Moreover, it can be based on the primer methods such as site-finding PCR [26], thermal asymmetric interlaced (TAIL) PCR [17], semi-random primer PCR [10], and linear and exponential TAIL (LE-TAIL) PCR [12]. Flanking-sequence exponential anchored (FLEA) PCR [20] uses random and degenerates primers with gene-specific primers [11, 33]. Single long primer PCR (SLRA PCR) used gene-specific primers and a random amplified polymorphic DNA primer for genome walking [15], and the stepwise partially overlapping primer (SWPOP-POP) method is a partial overlap of the latter primer that is identical to the 5′ part of the former one [2].

The restriction base method of genome walking requires a primitive digestion of genomic DNA by restriction enzymes. This digestion should be located at an appropriate distance between unknown and known regions [14]. An enzyme with a restriction site at a distance away from a gene-specific primer, not too far in order to allow subsequent PCR amplification, is valuable. Sometimes it is difficult to predict the correct choice of the restriction enzyme due to the lack of sufficient information about sequences [21]. Restriction fragment can be subsequently either self-circularized or ligated to the designed adaptors. These adaptors are created from the modification of DNA termini and/or double-strand cassette for connection to the genome [14]. T-linkers are examples of the first case that is caused by the modification of DNA termini [34]. Adaptors that are ligated separately to the genome fragments are double-stranded adaptor with a blunt end. In this adaptor, a shorter strand is blocked with an amine group [24]. Other double-strand adaptors are vectorette adaptor (double-strand adaptor with a mismatch region in the center) [1], spelinkerett adaptor (the same as the vectorette adaptor but with a hairpin structure) [7], adaptor consisting of a hairpin structure and tail-A (hairpin structure and tail-A ligated with phosphorothioate linkage) [18], and phosphorylated excess base adaptor (double-strand adaptor with sticky end and one base excess) [28]. Besides the large application of genome walking, sometimes, availability, cost of adaptors, and importation of genome walking kits are the barriers to the researcher’s performance.

Recently, several protocols have been developed using the combination of next-generation sequencing (NGS) technologies and genome walking. These protocols are used to study the insertion mechanisms of retroviruses, gene therapy, and functional genomics [31]. In virtual genome walking, gene models have been generated with low Illumina data, and algorithms can be used to walk a high number of repeats [8]. This method (Illumina) has been used for comparative analysis of the chloroplast genome of *Stryphnodendron adstringens* with related *Mimosoid* species [5].

There are more than 53 types of modification strategies to perform genome walking technique. Each of these methods is achieved by designing various primers (random primer, specific primer, etc.), ligation reaction step, and adaptors. Some of these methods do not have enough efficiency to identify the unknown nucleotide sequences and confirm PCR products. In addition, some methods have problems such as low precision for genome walking because of low specificity, high cost, being time-consuming, and limitations in performing genome walking in different sequences. In the restriction-based method of genome walking, just a few restriction sites are considered on the adaptors of common genome walking kits. This can also limit the researchers’ choice of a suitable enzyme for the desired genome. Also, the adaptor should be modified by adding 5′ phosphate and 3′ amine groups for the ligation process which increases the handling costs.

Restriction enzymes cut a double-strand DNA, so in this research, a double-strand adaptor with multiple restriction sites has been designed. Therefore, it is possible to select the desired enzyme for the genome, or researchers are able to substitute their desired restriction sites instead of the present adaptors’ multiple restriction sites. Additionally, since restriction enzyme digestion in double-strand adaptor provides the 5′ phosphate group, it is not required to add phosphate, amine, or other groups to the adaptor after digestion, and digested adaptor containing 5′ phosphate group can be ready for ligation with any DNA fragment without modification. In the present study, this strategy has been investigated by convenient PCR for the identification of *Momordica charantia* target fragment as a model. In addition, correct performance of adaptor was investigated using pTZ57R plasmid sequence. The availability of whole pTZ57R genome sequence allowed to accurately determine the performance of the designed double-strand adaptor.

## Methods

### DNA isolation and restriction digestion

*Momordica charantia* L. (bitter melon, known as karela) commercial seeds (East-West Seed, PALEE F1) were purchased from Durga Seeds, Pakistan, and were grown at a growth chamber (25 °C and 16-h light) laboratory conditions. Genomic DNA was isolated from the young leaves of *Momordica charantia* by the modified Dellaporta method [6]. First, 0.2 g of plant material was grounded in liquid N_2_ and transferred to the tube, then 600 μL extraction buffer (100 mM Tris, 50 mM EDTA, 500 mM NaCl, 1% SDS, and 1 μL RNase A 10 mg/mL) was added to the tube and incubated at 65 °C for 15 min. The tube was centrifuged (Eppendorf, Germany) at 17,000*g* for 10 min. The supernatant was transferred to a new tube and an equal volume of 1:1 phenol/chloroform and 5 M potassium acetate was added. The tube was centrifuged at 17,000*g* for 10 min and the supernatant was transferred to a new Eppendorf tube, and an equal volume of cold isopropanol was added. The tube content was mixed and incubated at − 20 °C for 30 min and subsequently centrifuged at 17,000*g* for 10 min. The supernatant was discarded, and the tube was washed with ethanol. The tube was centrifuged at 17,000g for 5 min, and the supernatant was discarded. After drying the tube containing sediments (DNA), 30 μL of deionized water was added to the tube and stored at − 20 °C. The DNA concentrations were determined using a spectrophotometer (BioPhotometer Plus, Eppendorf, Germany) at 260 nm, and DNA quality was determined by agarose gel electrophoresis. Application of the partial digestion is more likely to achieve a large sequence as a template in genome walking; therefore, *Hind* III, *Bam*H I, *Eco*R I, and *Bgl* II enzymes with no restriction site in a known sequence of the *map30* gene were selected, and the best digestion of DNA was achieved for the *Bgl* II enzyme. Digestion was performed using 1 μg of genomic DNA and 1 μL of the enzyme by incubating at 37 °C for 16 h. Digested DNA was purified with phenol/chloroform (1:1 v/v) extraction and then was precipitated by an aqueous solution of 70% ethanol as described by Sambrook et al. [23].

### Construction of oligonucleotides

**Fig. 1:**
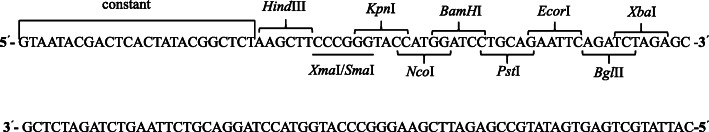
Structure of adaptor consists of two complementary oligos that comprise ten restriction sites

A double-stranded adaptor was constructed by annealing of two unphosphorylated cassettes with 70-bp nucleotides. Each cassette consists of a constant and a variable segment. There are 10 restriction sites with different restriction patterns that phosphorylated when digested (Fig. 1). Adaptor-specific primers were designed on a constant segment. Annealing was performed by heating the cassettes (10 μM) in a water bath (Memmert, USA) at 95 °C for 5 min. Then, the cassettes were slowly cooled to room temperature.

### Primers

Three specific primers (GSP1 and GSP2 as reverse primers and F helper as forward primer) of a gene sequence are required in the reaction for amplification of flanking sequences. The location of the external (GSP1) and internal (GSP2) primers are considered at a distance of about 150–250 nucleotides from the beginning of the gene. F helper was designed based on the first known nucleotide sequences of the forward primer. The quality of the designed primers was checked by the OligoAnalyzer tool of Integrated DNA Technologies available at http://www.IDTDNA.com/calc/analyzer. Two adaptor primers AP1 and AP2 (forward primers) were designed based on the constant cassette of the adaptor. Primers were synthesized by Macrogen Company, South Korea. The list of primers is available in Table 1.

**Table 1:** Primers used in this study

Primers name	Primer sequence (5′-3′)	Location	Primer application	*T*_*m*_ (°C)
GSP1	GCATAACTTGTGAGATTGAGG	*map30* gene	Reverse primer in the 1st PCR	56.5
AP1	GTAATACGACTCACTATACGGC	Adaptor	Adaptor primer in the 1st PCR	56.5
GSP2	GGCTAAATGGAAGAGTCG	*map30* gene	Reverse primer in the 2nd PCR	54
AP2	GACTCACTATACGGCTCT	Adaptor	Adaptor primer in the 2nd PCR	54
F helper	GCATGGTGAAATGCTTACTAC	*map30* gene	Forward primer in the 3rd PCR	50.5

### Ligation

Genomic DNA and adaptor were digested by *Bgl* II (Thermo Fisher). The same sticky end in genomic DNA and adaptor were created for the ligation reaction. The digested products were evaluated on 2% agarose (Sigma-Aldrich, USA) gel. The gel was stained with ethidium bromide (Sigma-Aldrich, USA) and visualized using UV doc (UviTec, Iran). Ligation was done using 1 μg of digested DNA, 0.5 μL of the digested adaptor, 1u of T4 DNA ligase enzyme (Thermo Fisher), and 2 μL of 10× ligase buffer in a total volume of 20 μL. The ligation reaction was incubated at 22 °C for 1 h. Finally, 9 μL of autoclaved ddH2O was added to 1 μL of ligation to make a 1:10 dilute solution for the first PCR.

### PCR amplification

The primary PCR reaction was carried out in a total volume of 15 μL. PCR mixture contained 0.5 μL of each of the forward (AP1) and reverse (GSP1) primers, 0.5–1 μg of diluted ligation product, and 8 μL Taq Master mix (Ampliqon, Denmark). The PCR was performed in a thermal cycler (Eppendorf Master Cycler Gradient, Germany) under the following conditions: predenaturation at 95 °C for 5 min followed by 30 cycles of denaturation at 95 °C for 1 min, annealing at 56.5 °C for 45 s, extension at 72 °C for 1 min, and a final extension step at 72 °C for 5 min. The second PCR was performed under the same conditions as the primary PCR using reverse (GSP2) and forward (AP2) primers and the first PCR products as a template with an annealing temperature of 54 °C. Finally, the third PCR was performed to confirm the presence of *map30* gene in the product. Two microliters of the second PCR product and reverse (GSP1) and forward (F Helper) primers was used in this PCR.

### Investigation of the adaptor performance

**Fig. 2:**
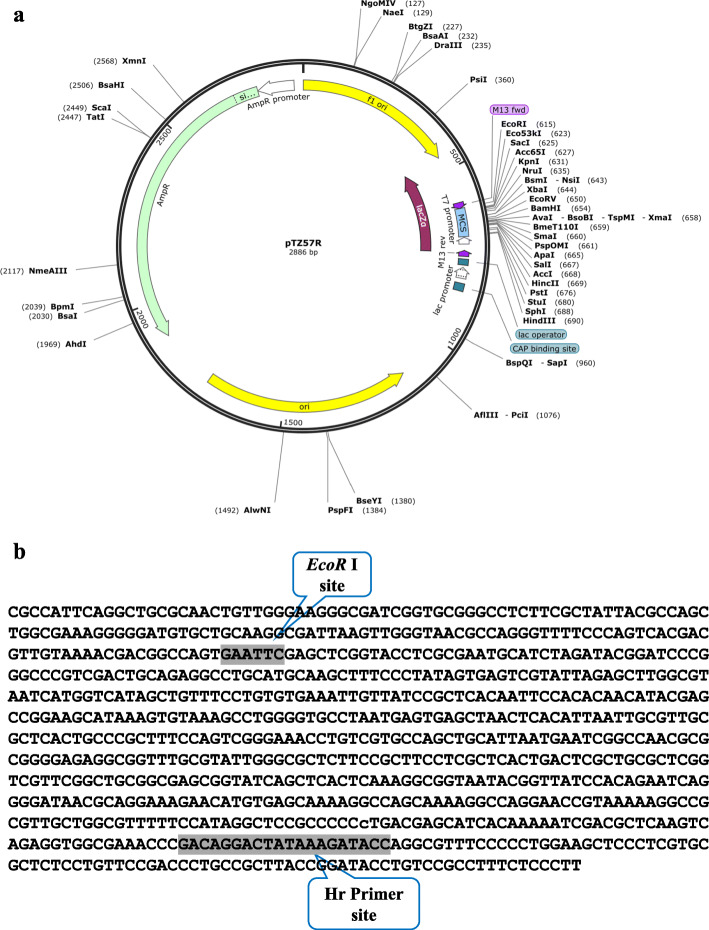
pTZ57R plasmid. **a** Map of plasmid and *EcoR* I restriction site location. **b** Partial sequence of the plasmid indicating location of *EcoR* I restriction site sequence and Hr-specific reverse primer

The plasmid DNA (pTZ57R) was subsequently used as a template to determine the reliance of adaptor performance in different sequences. First, *EcoR* I restriction enzyme was selected among the available restriction sites of the double-strand adaptor. The double-strand adaptor and pTZ57R plasmid sequence were digested by *EcoR* I to generate sticky ends (partial digestion) (Fig. 2a). Then, ligation reaction using the phosphorylated terminal adaptor and the digested plasmid was done with the T4 ligase enzyme. Eventually, PCR was performed using the AP1 adaptor primer as a forward primer on adaptor and gene-specific Hr primer (5′-GGT ATC TTT ATA GTC CTG TCG G-3′) as a reverse primer at a distance of about 600 nucleotides from the *EcoR* I site in the plasmid (Fig. 2b).

## Results

**Fig. 3:**
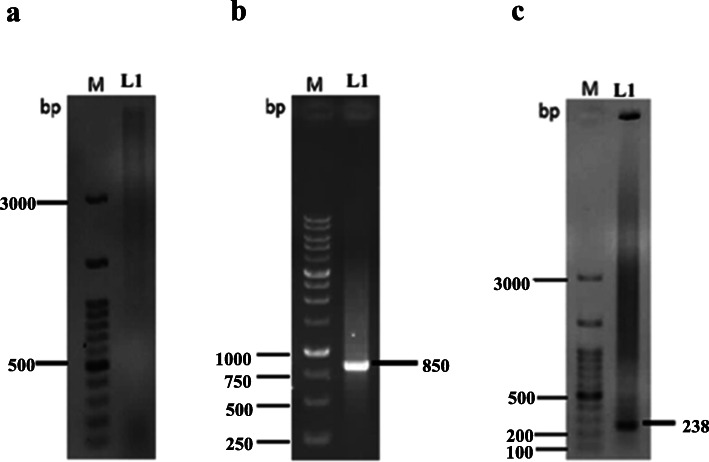
Amplification of PCR fragments using different primers. **a** GSP1 and AP1 primers in the first PCR. M, DNA ladder; L1, first PCR product amplified from the upstream region of *map30* gene. **b** GSP2 and AP2 (nested primer) primers in the second PCR. M, DNA ladder; L1, second PCR product amplified from the upstream region of *map30* gene. **c** GSP1 and F helper primers. M, DNA ladder; L1, third PCR product

The double-stranded adaptor was digested by *Bgl* II restriction enzyme to generate sticky ends. Naturally, after digesting the adaptor, phosphorylated 5′ overhang was made. In the next step, genomic DNA was digested with the same enzyme, followed by the ligation reaction between the phosphorylated terminal adaptor and the digested DNA using a T4 ligase enzyme. Performing two PCR reactions was necessary for this method. In the first cycle of primary PCR, only the gene-specific primer (GSP1as reverse primer) binds to *map30* sequence followed by making ssDNA. This will provide a complementary strand for the adaptor primer site (AP1as forward primer) in the next step of PCR. In the first PCR, the copy number of upstream gene sequence increased, but products were shown as a smear on agarose gel electrophoresis (Fig. 3a). Two microliters of the first PCR products was subjected as a template in nested PCR. The role of GSP2 (reverse) and AP2 (forward) primers is in the second PCR. The amplicon with a size of about 850 bp, the upstream region of the *map30* gene, was obtained (Fig. 3b).

According to the results of this study, it is suggested to perform the third PCR for confirmation of target sequencing results. If non-specific PCR products are observed on an agarose gel, the third PCR could be performed with gene-specific primer and F helper (forward helper) for the final confirmation of the results. F helper site is at the beginning of the *map30* gene, and its distance with the gene-specific primer is 238 bp (Fig. 3c).

The upstream regions of the *map30* gene are unknown. Hence, the PCR product was sent for sequencing to the Macrogen Company (South Korea). TATA box was determined using PLANT CARE bioinformatics software. Moreover, *cis*-acting regulatory elements, light-responsive elements, gibberellin-responsive elements, and MYB binding site were identified.

**Fig. 4:**
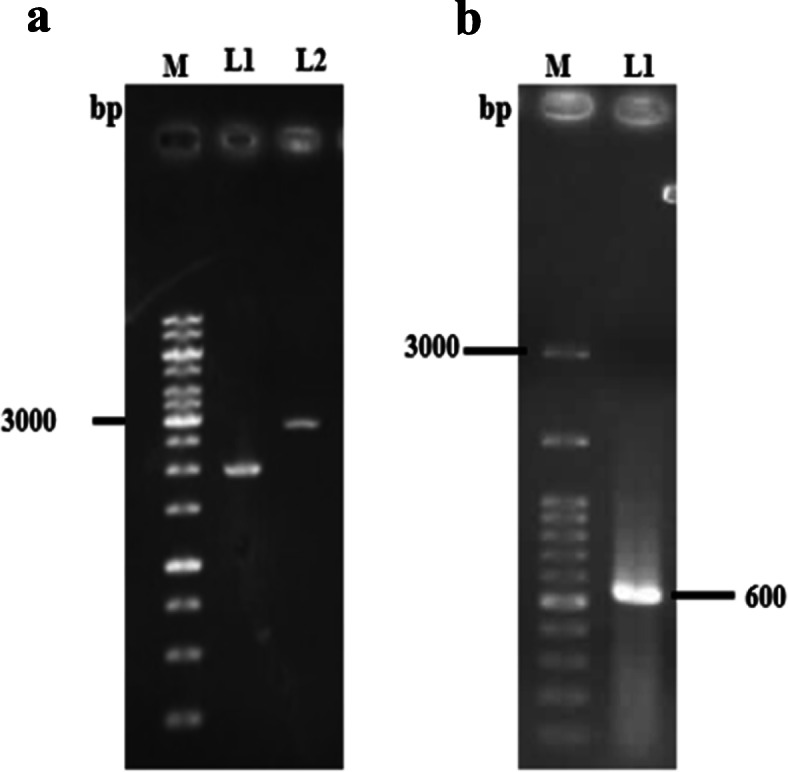
Assessment of adaptor performance using pTZ57R plasmid. **a** M, DNA ladder; L1, intact supercoil plasmid; L2, digested plasmid with *EcoR* I. **b** M, DNA ladder; L1, amplified 600-bp PCR product of AP1 (forward) and Hr (reverse) primers

The performance of the double-stranded adaptor was achieved by pTZ57R plasmid. Figure 4a indicated the intact supercoil form of pTZ57R and digested plasmid with *EcoR* I restriction enzyme. The result of PCR showed the 600-bp fragment, amplified by AP1 (forward) and Hr (reverse) primers (Fig. 4b).

## Discussion

**Fig. 5:**
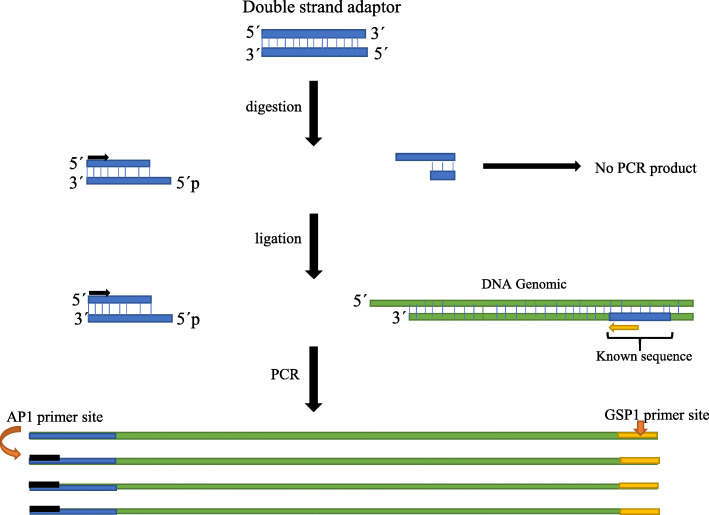
The schematic diagram of PCR indicating the position of the adaptor primer and phosphate group followed by ligation reaction; the double-strand blue adaptor and genomic DNA digested with *Bgl* II enzyme. 5′ phosphate group sticky end creates in adaptor and DNA. After ligation, the first GSP1 primer (yellow arrow) binds to genomic DNA, extends ssDNA, and provides a site to anneal AP1 (black arrow). The short adaptor fragment does not have the primer site, and it cannot be as a template. In the next steps, PCR extends the desire product

The present study was conducted to design a simple double-strand genome walker adaptor. This adaptor does not require modifications such as the addition of a nucleotide or phosphate group. Each of the restriction enzyme site can be phosphorylated after in vitro digestion of the adaptor and genomic DNA. Digested site of the adaptor fragments does not have an adaptor primer site. Therefore, only fragments with adaptor primer sites will be extended, and this avoids the formation of non-specific fragments; hence, the method eliminated non-specific products (Fig. 5). In the restriction-based genome walking technique, it is important to obtain a proper length sequence to gain more information from unknown regions. In 2012, choosing the proper restriction enzyme to develop genome walking and characterize flanking DNA is focused. They digested the whole genome in silico digestion for selecting restriction enzymes. Furthermore, they calculated the frequency and average fragments for each restriction enzyme [25]. However, this approach can only be followed for species with available whole-genome sequences.

We designed an adaptor with 10 different restriction sites, so that if one restriction enzyme is not able to digest the sequence, another enzyme can be applied. Therefore, this approach allows the researchers to have appropriate sizes of products without any limitations in the selection of restriction enzymes (3′ overhang, 5′ overhang, blunt end). Moreover, by the present method, the researchers can substitute their desired enzymes instead of adaptors’ restriction site enzymes. In this study, it is demonstrated that convenient PCR combined with this adaptor and specific primers can help to obtain novel genome walking.

Partial digestion of genomic DNA helps to have longer fragments in genome walking, but in our case, the distance between *Bgl* II restriction site and gene-specific primer in the genome was not too far. In the second PCR, amplified fragments from ligation were about 850 bp on an agarose gel.

The digested genome should properly be ligated to the adaptor (the digested adaptor and genome have the same end). In T-linker PCR, despite the extension of the sequence, due to unphosphorylated T-linker, there is a nick between chromosomal DNA and the ligation T-linker. On the other hand, T-linker PCR requires 3–6 enzymes (3′ overhang restriction enzymes) that can be limited on the selection of enzyme [34], while the adaptor designed in this study, by creating a phosphate group after digestion with a suitable enzyme, eliminates the nick between the adaptor and the genomic DNA.

Not only do adaptor and special primers designed in this study have many advantages over the use of random degenerate primers, they can also be used to confirm each of the obtained sequences with no requirement of performing semi-random PCR [10] or cloning the products. In some methods, palindromic sequence-targeted PCR (PST-PCR) degenerate sequence (8–12 nucleotides long) is used; hence, multiple products may be observed after PCR with primers or products not observed for some primers, and researchers have to redesign primers in different locations [13]. In our optimized method, an adaptor with more enzyme restriction site and specific primer based on the first part of the adaptor sequence is designed; hence, the possibility of unwanted fragment amplification is reduced, therefore making the method more convenient compared to the PST-PCR and traditional genome walking methods.

## Conclusion

In this research, a double-strand adaptor with multiple restriction sites has been designed. This strategy has been investigated by convenient PCR to identify the target. The accomplishment of the genome walking using this method reduced the time and cost of study by designing specific primers and simple adaptor. To confirm the target sequence between multiple possible products, one PCR (third PCR) was performed instead of cloning or redesigning specific primers. To obtain longer flanking DNA, another restriction enzymes or high-fidelity DNA polymerase could be used. Overall, the synthetic adaptor sequence and forward primer designed based on the adaptor could be used to identify the unknown region of any gene. The users of the method only need to design a reverse primer using a known sequence of the target gene. In addition, the application of the present method helps researchers to substitute their desired restriction enzyme sites instead of the present adaptors’ multiple restriction sites.

## Data Availability

The datasets used and/or analyzed during the current study are available from the corresponding author on reasonable request.

## Abbreviations

PCR: Polypeptide chain reaction
GW: Genome walking
RAGE: Rapid amplification of genomics ends
TAIL: Thermal asymmetric interlaced
LETAIL: Linear and exponential TAIL
FLEA: Flanking-sequence exponential anchored
NGS: Next-generation sequencing
EDTA: Ethylene diamine tetraacetic acid
SDS: Sodium dodecyl sulfate
GSP1: Gene-specific primer 1
GSP2: Gene-specific primer 2
F helper: Forward helper
AP1: Adaptor primer 1
AP2: Adaptor primer 2
